# Enrichment of Oil Cake With Cinnamon Extract Positively Effects Antioxidant Activity and Textural Profile

**DOI:** 10.1002/fsn3.4714

**Published:** 2025-03-04

**Authors:** Banafshe Bordbar Lomer, Fatemeh Ghannadiasl

**Affiliations:** ^1^ Department of Food Science and Technology Urmia University Urmia Iran; ^2^ Department of Food Sciences and Technology University of Mohaghegh Ardabili Ardabil Iran

**Keywords:** antioxidant activity, aqueous cinnamon extract, *Cinnamomum zeylanicum*, oil cake, texture profile analysis

## Abstract

Identifying the optimal formulation is essential for achieving health benefits, preserving texture, and enhancing the flavor of baked goods. This study examined the effects of aqueous cinnamon extract ( *Cinnamomum zeylanicum* ) at concentrations of 0.05%, 0.10%, 0.15%, 0.20%, and 0.25% (W/V) on the antioxidant activity, physicochemical, textural, and sensory properties of oil cakes enriched with these extracts. HPLC analysis of the aqueous extract identified cinnamaldehyde as the dominant compound (30.5%), along with significant amounts of eugenol, cinnamic acid, and coumarin. The results showed that increasing the cinnamon extract concentration enhanced TPC, TTC, %RSA, and %FRAP values. The pH of the cake samples did not significantly differ across concentrations (*p* < 0.05). The moisture content was higher than the control, but water activity decreased with higher extract concentrations. The 0.25% sample showed significant differences in protein content compared to the control, 0.05%, and 0.10% samples (*p* < 0.05). Fat and carbohydrate contents were generally lower than the control. TPA results showed decreased hardness, cohesiveness, resilience, and fracturability with higher cinnamon extract levels. Additionally, increased extract levels improved the springiness of the cakes. The 0.20% and 0.25% samples had the highest overall consumer acceptability. The study found that samples containing 0.20% and 0.25% cinnamon extract were the most effective concentrations for oil cakes. This indicates that aqueous cinnamon extract, with its antioxidant properties, can serve as a beneficial additive to enhance the quality of oil cakes.

## Graphical abstract

This study shows that incorporating aqueous cinnamon extract into oil cakes improves antioxidant activity, texture, and consumer acceptability. Concentrations of 0.20% and 0.25% significantly boosted total phenolic content and antioxidant capacity while enhancing texture and lowering fat and carbohydrate levels. Cinnamaldehyde, the main compound in the extract, contributed to these improvements, indicating that cinnamon extract is a valuable natural additive for enhancing the nutritional and sensory quality of oil cakes.

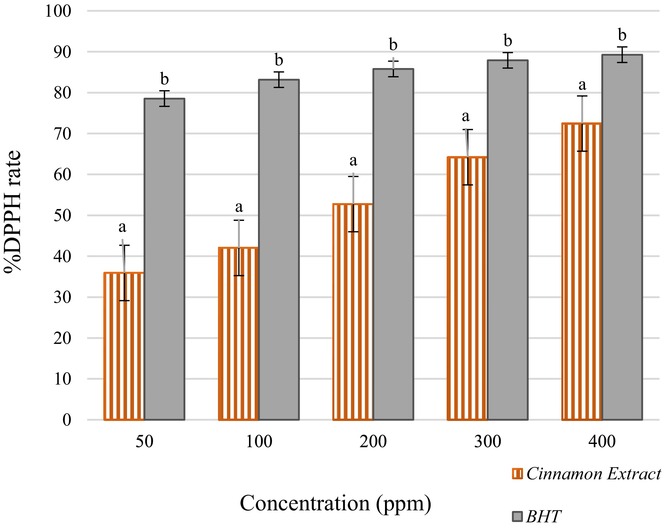

## 1 Introduction

Oil cakes are one of the most popular bakery products due to their excellent texture and taste, which are consumed by all age groups. Oil, as the main ingredient, is responsible for maintaining the gas cells in the oil/water emulsion, the volume, the softness, and the taste of the cake (Matsakidou, Blekas, and Paraskevopoulou 2010; Rios et al. 2014). It has been shown that free radicals react with lipids, causing lipid peroxidation in food, which leads to the production of mutagenic substances and changes in color, odor, texture, and quality of food (Esmaeilzadeh Kenari, Mohsenzadeh, and Amiri 2014). Additionally, oxidative stress produced by free radicals has been linked to cardiovascular disease, diabetes, hypertension, atherosclerosis, Parkinson's and Alzheimer's diseases, cancer, rheumatoid arthritis, and even accelerated nerve aging (Fitó, de la Torre, and Covas 2007; Sies, Berndt, and Jones 2017). As a result, there is a growing awareness among consumers about the potential dangers of oil, leading to increased efforts to mitigate its negative effects. Hence, natural or synthetic antioxidants are emphasized to protect against oxidative stress (Ismail et al. 2017; Rodriguez‐Sandoval, Prasca‐Sierra, and Hernandez 2017).

Due to the more practical effects and lack of side effects, there is a great interest in using natural antioxidants. For this reason, industry and researchers are always looking to optimize the technology for preparing these types of products, to improve the variety, quality, and taste (Dhillon and Amarjeet 2013; Şahin and Elhussein 2018). It is essential to create functional bakery products that are both physiologically beneficial and appealing to consumers in terms of appearance, taste, and texture. Therefore, research focused on the identification, extraction, purification, and application of antioxidants derived from natural sources is highly valued in the food industry (Ashfaq, Siddique, and Shahid 2021; Siró et al. 2008). When producing a range of high‐quality bakery products with significant nutritional value, enhancing sensory characteristics is a top priority for consumers. Therefore, determining the effective formulation that promotes health benefits, preserves texture, and improves the flavor of these products is crucial (Škrbić and Cvejanov 2011).

The nutritional and medicinal value of plants is well known. *Cinnamon zeylanicum*, a common type of cinnamon used as a nutraceutical, belongs to the *Lauraceous* family. This family contains a wide range of phytochemicals with antioxidative, and anti‐tumor activities, reducing the risk for cardiovascular diseases (Helal and Tagliazucchi 2018; Khalisyaseen and Mohammed 2021). It contains procyanidins and catechins with high antioxidant activities. Consequently, assessing the concentration of phytochemicals present in the extract of this plant is very important (Rao and Gan 2014; Sivapriya and John 2016). Cinnamon powder is a well‐known and widely used traditional herbal remedy in the Middle East and Arab countries. The main components in cinnamon bark are phenolic compounds and cinnamaldehyde, which exhibit various biological activities such as anti‐tumor, pro‐apoptotic, and anti‐inflammatory properties (Helal and Tagliazucchi 2018).

Cinnamon has been used for flavoring, coloring, or preservatives in various food products (Saleem et al. 2015). In the study by Murcia et al. (2004), cinnamon was found to be the best superoxide radical scavenger compared to Roman anise, ginger, licorice, nutmeg, and vanilla. Another study concluded that cinnamon extract possesses significant antioxidant properties and may serve as a natural alternative to synthetic antioxidants for enhancing human health and nutrition (Shahid et al. 2018). In the study carried out by Hiregoudar, Revanna, and Mamatha (2023), it was mentioned that further research on the commercial production of cakes using cinnamon essential oil is necessary. This essential oil not only acts as a natural preservative but also as a functional ingredient in bakery products (Hiregoudar, Revanna, and Mamatha 2023). The objective of this research was to investigate the antioxidant and physicochemical properties of different concentrations of cinnamon extract in oil cake, as well as to evaluate textural and sensory characteristics.

## 2 Materials and Methods

### 2.1 Preparation of Cinnamon Extract

To prepare the extract, samples of cinnamon bark ( *Cinnamomum zeylanicum* , Golestan brand, Kian Badas Company of Tehran) were purchased and pulverized using an electric grinder (France Moulinex Company, A320R1) under suitable conditions and away from sunlight. To prepare an aqueous extract, the resulting powder was soaked in distilled water for 48 h at 25°C (using 300 mL of distilled water as a solvent for every 100 g of powder). The soaked powder was transferred to a Buchner funnel and filtered using Whatman No. 42 filter paper. The suspension was then centrifuged for 15 min at 5000 rpm to separate the solids from the solution. The resulting extract was concentrated by a rotary evaporator (EV400‐Labtech‐Italy) at a temperature of 75°C and a speed of 200 rpm in a vacuum until it reached 15°Brix. Brix measurements were performed with a refractometer (Onderoglu et al. 1999). The resulting extract was then used to prepare the desired concentrations (0.05%, 0.10%, 0.15%, 0.20%, and 0.25% W/V). It should be noted that this extract was stored in a dark glass container with a closed lid and kept in a refrigerator at 4°C until further use.

### 2.2 HPLC Analysis of Aqueous Extract of Cinnamon

The high‐performance liquid chromatography (HPLC) chromatogram (Waldbronn, Germany) of the aqueous extract of *Cinnamomum zeylanicum* was obtained using a C18 column with a gradient elution of water (0.1% formic acid) and acetonitrile (HPLC grade). The flow rate was set at 1.0 mL/min, and detection was carried out at a wavelength of 280 nm.

To prepare the cinnamon bark for analysis, it was finely ground using a mortar and pestle to ensure a sufficient amount of plant material. Dry cinnamon samples weighing 0.5 g were precisely measured and then sonicated in 20 mL of 80% methanol (HPLC grade) for 30 min. After sonication, the mixture was centrifuged at 3000 rpm for 10 min. The resulting supernatant was carefully transferred to a 25‐mL volumetric flask. This extraction process was repeated with an additional 5 mL of 80% methanol, and the combined supernatants were brought to a final volume of 25 mL using methanol. The solution was thoroughly mixed to ensure homogeneity. Prior to injection, about 2 mL of the solution was filtered through a 0.2‐μm nylon membrane filter, discarding the first 1 mL of filtrate. The remaining filtrate was collected in an LC sample vial for further analysis (Al‐Zehouri 2017).

### 2.3 Antioxidant Activity of Cinnamon Extract

Antioxidant activity is primarily dependent on the availability of electrons to neutralize free radicals (Saber 2019). It has been shown that cinnamon bark contains a high concentration of antioxidants (Haddi, Faroni, and Oliveira 2017). In this study, commonly used methods such as the measuring total phenolic compounds (TPC), total tocopherol compounds (TTC), Diphenyl picrylhydrazyl (DPPH), and ferric reducing antioxidant power assay (FRAP) were employed to assess the antioxidant capacity of food compounds in a laboratory setting.

#### 2.3.1 Measurement of TPC

The TPC in the cinnamon extract was determined using spectrophotometry with the Folin–Ciocalteu reagent (Delfanian, Esmaeilzadeh Kenari, and Sahari 2015). In this method, the Folin reagent is reduced in the presence of phenolic compounds, resulting in the formation of a blue dye in an alkaline environment. For this purpose, 0.5 mL of the extract was diluted with 2.5 mL of 10% Folin–Ciocalteu reagent (diluted 10 times), and then 2 mL of 7.5% sodium carbonate solution (75 g/L) was added. The mixture was shaken for 1 min and left in the dark for 15 min at room temperature. The absorbance of the solution was measured at 764 nm using a spectrophotometer (UV‐M51, UV/VIS Spectrophotometer, Italy). All tests were performed in triplicate.

The absorbance values were converted into total phenolic content (TPC) utilizing the calibration curve created with the gallic acid standard. The TPC was expressed as mg GAE/mL. The calibration curve had an *R* ^2^ value of 0.9967 and the equation of the line was *Y* = 0.0151 *X* + 0.0355.

#### 2.3.2 Measurement of TTC

The amount of TTC in the cinnamon extract was measured based on alpha‐tocopherol. For this purpose, 1 mL of the extract with different concentrations was mixed with 5 mL of toluene, 3.5 mL of 2′‐2 bipyridine solution (0.07% w/v in 95% aqueous ethanol), and 0.5 mL of FeCl_3_‐6H_2_O (0.2% w/v in 95% aqueous ethanol). The absorbance was then measured at a wavelength of 520 nm using a spectrophotometer (UV‐M51, UV/VIS Spectrophotometer, Italy) after 1 min (Saviz, EsmaeilzadehKenari, and KhalilzadehKelagar 2015).

#### 2.3.3 DPPH Free Radical Scavenging Activity

Measurement of free radical scavenging activity (RSA) of DPPH was performed using a spectrophotometric method at a wavelength of 517 nm. In this method, 0.3 mL of the extracts at different concentrations were added to 2.7 mL of DPPH solution (6 × 10^−5^ mol/L) and kept in the dark for 60 min. Finally, the absorbance of the solutions was measured using a spectrophotometer (UV‐M51, UV/VIS Spectrophotometer, Italy) at 517 nm with a distilled water blank. The %RSA of DPPH was calculated using the following formula (1):

(1) $$I\%=\left(A_{B}-A_{S}\right)/A_{B}\times 100$$

In the formula, *A*_S_ represents the absorbance of the sample at different extract concentrations, and *A*_B_ is the absorbance of the control sample, measured after 60 min. By plotting the graph of the percentage of RSA against the concentration of the antioxidant compound, the best‐fit line equation is determined for the points, and then IC_50_ is calculated (Esmaeilzadeh Kenari, Mohsenzadeh, and Amiri 2014). Also, the synthetic antioxidant butylated hydroxytoluene (BHT) was used as a reference to compare the antioxidant activity of the extract.

#### 2.3.4 Measurement of FRAP

2.5 mL of the cinnamon extract was added and incubated with 2.5 mL of 20 mM phosphate buffer solution (pH = 6.6) and 2.5 mL of 1% potassium ferricyanide. Then 2.5 mL of 10% w/v trichloroacetic acid was added and the mixture was centrifuged (Hettich ZENTRIFUGEN, Germany/Model D‐7200 Tuttlingen). The absorbance values of the samples were then read at 700 nm (Esmaeilzadeh Kenari, Mohsenzadeh, and Amiri 2014). A methanolic solution of Tertiary Butylhydroquinone (TBHQ) was utilized as the standard.

### 2.4 Preparation of Cake

The studied samples included a control sample (without extract) and samples enriched with five different concentrations of cinnamon bark extract (0.05%, 0.10%, 0.15%, 0.20%, and 0.25% W/V), which were incorporated during the preparation of the cake dough. The cake was made using the sugar‐dough method. In the first step, the oil (263 g) and sugar (330 g) were heated until a light color was achieved (about 10 min). Then, the eggs (330 g) were added in several portions. Next, all the powdered ingredients were sifted (flour 425.6 g, baking powder 7.5 g, milk powder 9.2 g, vanilla 2.3 g, and whey powder 18.4 g), and then added to form a semi‐uniform dough. Water was also added as needed to achieve the desired dough consistency. The prepared dough was transferred into muffin molds and baked in an oven set to 170°C for 25 min. Afterward, the cakes were allowed to cool at room temperature for 30 min, then packaged in polyethylene bags and kept at room temperature until the experiments were conducted (Peyghambardoust 2010).

To assess the antioxidant activity of the cake samples, 1 mL of distilled water was added for every 0.1 g of the cake samples. The mixture was shaken at a speed of 200 rpm for 2 h. Then, the samples were centrifuged at 5000 rpm for 5 min. The supernatant was separated using Whatman No. 42 filter paper, and the obtained filtrate was utilized for the analysis (Pasqualone et al. 2014).

### 2.5 Antioxidant Activity of Oil Cake

To assess the antioxidant activity of fresh oil cake samples, 1 mL of distilled water was added for every 0.1 g of the cake sample. The mixture was shaken at 200 rpm for 2 h and then centrifuged at 5000 rpm for 5 min. The supernatant was filtered using Whatman No. 42 filter paper, and the filtrate obtained was used for the experiment (Pasqualone et al. 2014).

### 2.6 Physicochemical Properties

The pH of cake samples dried in the oven at 45°C for 12 h was measured after preparing the cake extract with distilled water at a ratio of 1:2, shaking at 200 rpm, and centrifuging at 5000 rpm for 2 h. The pH was finally measured using a desktop pH meter (827 pH Lab—Metrohm, Switzerland), following AOAC method No. 945/10 (AOAC 1990). The moisture content of the fresh cake samples was evaluated according to the guidelines of Iranian national standard No. 2705 (ISIRI 2010). Additionally, the water activity of the cake samples was assessed at 25°C using a water activity meter (Novasina Labmaster neo, Switzerland), based on Iranian national standard No. 2553 (ISIRI 2021). The protein content was determined using the Kjeldahl method (Gerhardt, KJELDATHERM, Germany), in accordance with Iranian national standard No. 19052 (ISIRI 2014). Fat content was obtained by a gravimetric method using soxhlet extraction (Behr Labor‐Technik, Germany) with hexane as the extraction solvent, based on AOAC method No. 920.39. Carbohydrate content was determined using the Lane‐Eynon method in conjunction with Fehling's titration technique, in accordance with Iranian national standard No. 2553. The total ash content was measured by incinerating the samples in a furnace (Heraeus, Germany) at 550°C. The acid‐insoluble ash content was then determined by adding 25 mL of 5 N hydrochloric acid to the raw ash, following the procedures outlined in Iranian national standard No. 37 (ISIRI 2024).

### 2.7 Texture Profile Analysis (TPA)

The TPA of the cake samples was performed using a Texture Analyzer Meter (Brook field‐CT310K model). The TPA is a force‐time curve that quantifies the texture properties (Vácha et al. 2014). To perform the TPA, oil cake samples with dimensions of 2 × 2 × 2 cm^3^ were compressed twice using a cylindrical probe with a diameter of 38.1 mm (probe code: TA4/1000) under the following conditions: test target 50%, trigger load 7 g, return speed 2 mm/s, test speed 2.00 mm/s, and pre‐test speed 1 mm/s. The TPA data were analyzed using Texture Expert 1.05 software. The measured parameters included hardness, adhesiveness, cohesiveness, resilience, fracturability, springiness, gumminess, and chewiness, with each sample replicated three times on average. Furthermore, the device was utilized to measure the dimensions of the cakes, including length, width, and depth.

### 2.8 Sensory Evaluation

The color, odor, taste, texture, and overall acceptability of cakes containing different percentages of cinnamon were evaluated by 20 trained food science students (12 women and 8 men, aged 22–26) in a quiet, well‐lit environment using a hedonic test. The volunteers were asked to rate these characteristics on a standard hedonic scale as described by Wichchukit and O'Mahony (2015), from 1 to 9. A score of 9 was assigned for highly desirable and excellent quality, and a score of 1 for highly undesirable and very poor quality (Wichchukit and O'Mahony 2015).

### 2.9 Statistical Analysis

In this study, all tests were conducted in triplicate. The effect of adding cinnamon extract to the oil cake was investigated using a factorial design in a completely randomized design and analyzed by SPSS software version 21.0. One‐way analysis of variance (ANOVA) and LSD post hoc test (*p* < 0.05) were used to compare group differences in various concentrations of cinnamon extract.

## 3 Results and Discussion

### 3.1 HPLC Analysis of Aqueous Extract of Cinnamon Extract

**FIGURE 1:**
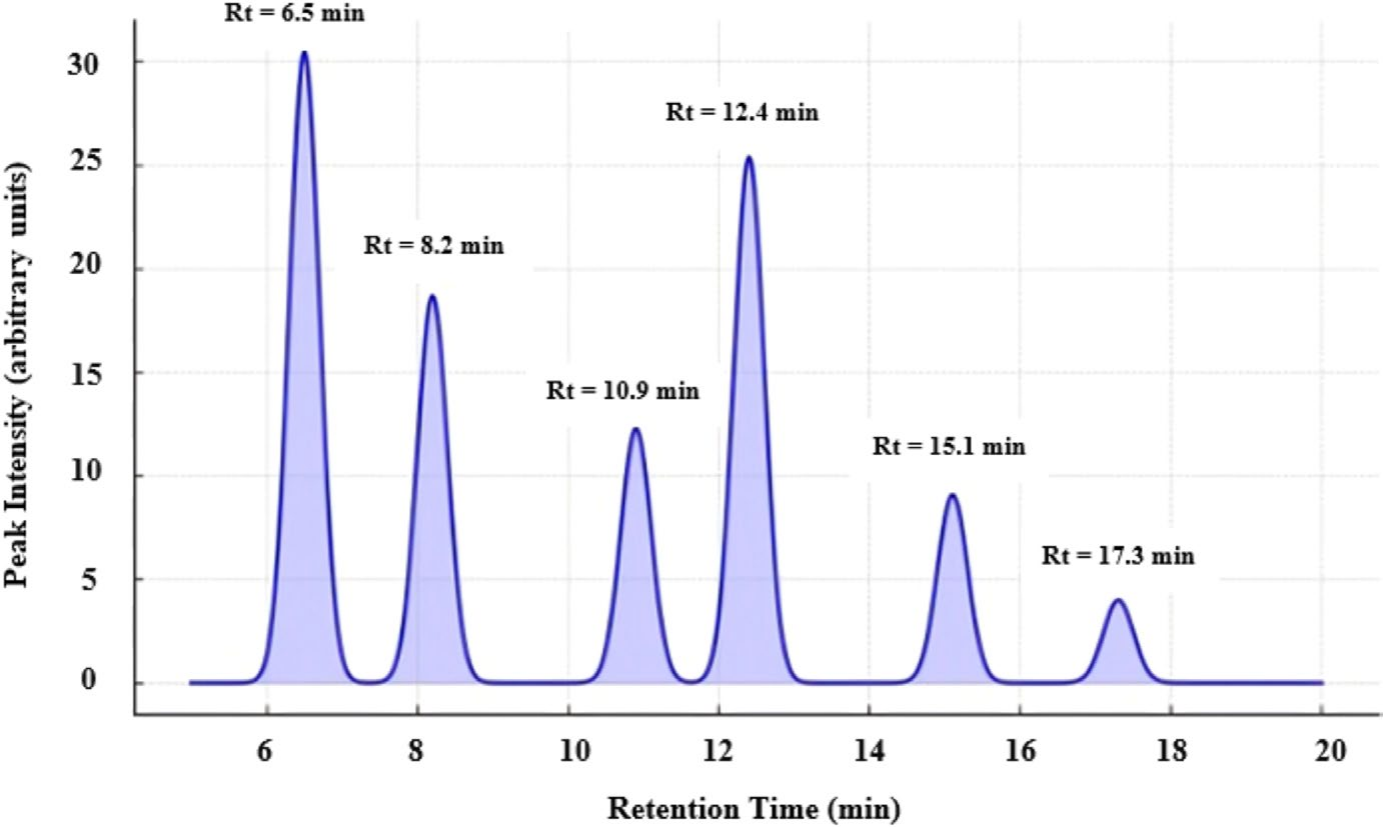
HPLC chromatogram of cinnamon aqueous extract. HPLC, high‐performance liquid chromatography.

The main compound detected in the aqueous extract was cinnamaldehyde, accounting for 30.5% of the total area under the curve, confirming its dominance in cinnamon (Figure 1). Significant quantities of eugenol, cinnamic acid, and coumarin were also observed. The chromatogram also displayed smaller peaks for other minor components (Table 1). The HPLC chromatogram for the aqueous extract of cinnamon bark ( *Cinnamomum zeylanicum* ) illustrates the retention time and peak intensity for the major identified compounds. The key components cinnamaldehyde, coumarin, eugenol, cinnamic acid, and catechin are marked with distinct peaks.

**TABLE 1:** HPLC peak identification and quantification of key compounds in cinnamon extract.

Peak no.	Compound	Retention time (min)	Area (%)
1	Cinnamaldehyde	6.5	30.5
2	Coumarin	8.2	18.7
3	Cinnamic acid	10.9	12.3
4	Eugenol	12.4	25.4
5	Catechins	15.1	9.1
6	Unidentified minor component	17.3	4.0

Jayaprakasha, Jagan Mohan Rao, and Sakariah (2003) identified the main compounds in cinnamon bark extract using HPLC as cinnamaldehyde, eugenol, and coumarin. Cinnamaldehyde was reported as the most abundant compound, contributing 45%–65% of the total area. This aligns with our findings where cinnamaldehyde is also the dominant peak at 30.5% (Jayaprakasha, Jagan Mohan Rao, and Sakariah 2003). The slightly lower concentration of cinnamaldehyde in our analysis compared to other studies may be due to differences in extraction methods (aqueous vs. ethanolic) and the source of cinnamon. In a study by Ranasinghe et al. (2013) on the aqueous extract of *Cinnamomum zeylanicum* , cinnamaldehyde was found to be the primary peak, followed by cinnamic acid and eugenol. The retention times were similar to our results, with cinnamaldehyde at ~6.0 min, cinnamic acid at ~10.5 min, and eugenol at ~12.0 min. The research indicated that the concentrations of cinnamaldehyde, cinnamic acid, and eugenol were 28%, 15%, and 24%, respectively, which closely align with our findings of 30.5%, 12.3%, and 25.4% (Ranasinghe et al. 2013). Another analysis of the essential oil components of cinnamon using HPLC by Singh et al. (2007) reported high concentrations of eugenol (up to 40%) and cinnamaldehyde (up to 50%) in volatile extracts. Eugenol tends to lower concentrations in aqueous extracts, due to its limited solubility in water. Our findings also support this with eugenol representing 25.4% of the peak area (Singh et al. 2007). The lower eugenol concentration in the aqueous extract indicates that solvent selectively extracts more water‐soluble components like cinnamic acid and coumarin compared to essential oil extraction methods.

The findings from our HPLC analysis align with previous research, particularly in identifying cinnamaldehyde as the main component in extracts of *Cinnamomum zeylanicum* . The relative proportions of cinnamaldehyde, eugenol, and cinnamic acid exhibit slight variations, likely due to differences in extraction techniques and sources of cinnamon. For instance, a study conducted by Yaseen and Mohammed found that aqueous extracts of *Cinnamomum zeylanicum* had significant quantities of phenols such as cinnamaldehyde and quercetin, both of which contribute to antioxidant activity (KhalisYaseen and Mohammed 2019). These results are similar to ours, with cinnamaldehyde as a significant component.

### 3.2 TPC and TTC of Cinnamon Extract

To determine the antioxidant potential of cinnamon bark ( *Cinnamomum zeylanicum* ), it is recommended to measure the antioxidant capacity or TPC (Klejdus and Kováčik 2016). Various extraction methods have been employed to extract phenolic compounds, extensive research focused on identifying efficient and environmentally friendly methods. The aqueous extraction method is notable for being cost‐effective, non‐toxic, environmentally friendly, and highly acceptable to consumers (González‐Centeno et al. 2015; Kallel et al. 2014).

In the study by Helal and Tagliazucchi (2018), the TPC extracted from cinnamon was reported to be 51.1 mg/g of cinnamon powder (Helal and Tagliazucchi 2018). Similarly, Klejdus and Kováčik (2016) found that after extraction with a 60% ethanol solution, the amount of phenolic compounds in cinnamon powder was 164 mg/g (Klejdus and Kováčik 2016). Another study by Qusti, El Rabey, and Balashram (2016) reported the phenolic compound content as 52.7 mg/100 g in methanolic cinnamon extract (Qusti, El Rabey, and Balashram 2016). Jayaprakasha and Rao (2011) calculated the phenolic compound content to be 44.5% in aqueous extract and 14.4% in ethyl acetate extract (Jayaprakasha and Rao 2011). Abeysekera, Premakumara, and Ratnasooriya (2013) reported TPC values of 33.43 mg gallic acid/g in ethanolic extract and 22.91 mg gallic acid/g in dichloromethane bark extracts of Ceylon cinnamon (Abeysekera, Premakumara, and Ratnasooriya 2013). Saleem et al. (2015) measured the TPC of cinnamon to be between 0.07 and 0.91 mg/g (Saleem et al. 2015). Muchuweti (2007) found the concentration of phenolic compounds to be 13.66 mg GAE in cinnamon methanolic extract (Muchuweti 2007). According to Saber (2019), the TPC in cinnamon powder was 8.21 mg/g, equivalent to gallic acid (Saber 2019). The amounts of TPC and TTC were evaluated using different extraction methods. Esmaeilzadeh (2019) and Sohrabpour (2019) reported the phenolic compound to be 29.95, 21, 30.35, and 6.63 mg/mL using ultrasound, supercritical fluid, solvent, and subcritical water extraction methods, respectively. The tocopherol compounds were measured at 20.17, 20.38, 10.95, and 20.86 mg/mL using the same methods (Esmaeilzadeh 2019; Sohrabpour 2019). Jamshidi, Barzegar, and Sahari (2014) also reported that the phenolic compound in cinnamon is 55.69 mg of gallic acid per gram (Jamshidi, Barzegar, and Sahari 2014). Overall, these studies indicate that cinnamon contains many phenolic compounds.

In the present study, the mean (±standard deviation) of TPC and TTC of the cinnamon extract were 25.51 (±0.0028) and 22.58 (±0.0058) mg/mL, respectively. Differences in the values obtained in various studies may be attributed to using different solvents or extraction methods (Esmaeilzadeh Kenari, Mohsenzadeh, and Amiri 2014). However, these studies consistently demonstrate that cinnamon contains a significant amount of phenolic compounds and possesses antioxidant properties, making it a potential additive in the food industry (Rao and Gan 2014).

### 3.3 DPPH Free Radical Scavenging Activity of Cinnamon Extract

After plotting the %RSA against different concentrations of cinnamon bark ( *Cinnamomum zeylanicum* ) extract, we obtained the best‐fit line equation for the points (*R* ^2^ = 0.99). The antioxidant capacity was expressed as an inhibitory concentration (IC_50_), which is the concentration of an antioxidant that inhibits 50% of the free radicals in the environment. Extracts with the lowest IC_50_ demonstrate the highest antioxidant properties (Esmaeilzadeh Kenari, Mohsenzadeh, and Amiri 2014).

According to the results of Table 2, the highest %RSA was observed at a concentration of 400 ppm, with the highest antioxidant properties (72.44%), while the lowest %RSA was at a concentration of 50 ppm, with the lowest antioxidant properties (35.91%). Increasing the concentration of cinnamon extract improves its ability to scavenge free radicals, thereby increasing the potency of the IC50 value. The reason could be that higher concentrations of cinnamon extract contain more bioactive compounds, such as cinnamaldehyde, eugenol, and polyphenols, which are responsible for neutralizing free radicals. At elevated concentrations (400 ppm), these compounds are present in sufficient amounts to demonstrate more significant antioxidant activity (72.44% RSA), in contrast to lower concentrations (50 ppm), where fewer bioactive compounds are available, resulting in reduced antioxidant activity (35.91% RSA). At elevated concentrations, these bioactive compounds may exhibit synergistic interactions, enhancing one another's antioxidant properties and consequently leading to a greater percentage of radical scavenging activity (%RSA). This result is consistent with another study investigating the antioxidant activity of sesame extract (Esmaeilzadeh Kenari, Mohsenzadeh, and Amiri 2014). The study by Xie et al. (2012) demonstrated that the concentration of the extract is an influential factor in increasing antioxidant activity. These results align with the findings of Nedamani et al. (2015), who found that increasing the concentration of rosemary and oak extracts enhances DPPH free radical inhibition power (Nedamani et al. 2015). The IC_50_ values for ethanolic, aqueous, and ethyl acetate extracts of cinnamon were reported at 8.36, 8.89, and 13.51 μg/mL, respectively (Ervina, Nawu, and Esar 2016). In another study, the DPPH values for ethanolic and dichloromethane extracts were 107.69 ± 2.01 and 60.49 ± 0.48 mg/g in cinnamon bark, respectively (Abeysekera, Premakumara, and Ratnasooriya 2013). The percentage inhibition of DPPH and IC_50_ of cinnamon powder was reported as 83.54% and 0.208 mg/mL, respectively (Saber 2019), which was higher than the %RSA obtained in our study (72.44%). The study of Vadivel and Brindha (2015) showed that the antioxidant activity obtained by the DPPH free radical inhibition test for garlic peel extract varied from 26% to 79% on average (Vadivel and Brindha 2015). The result obtained in this research was consistent with the %RSA obtained in our study (72.44%). In general, the differences in the antioxidant activities of plant extracts can be attributed to the different structures of plant extracts due to phenolic acids, flavonoid compounds, and their derivatives (Rababah, Hettiarachchy, and Horax 2004).

**TABLE 2:** IC_50_ values at different concentrations of cinnamon extract.

Concentration (ppm)	%RSA	IC_50_ (ppm)
50	35.91 ± 0.02^e^	3494.62 ± 0.82^a^
100	42.02 ± 0.01^d^	2546.75 ± 0.86^b^
200	52.74 ± 0.09^c^	1598.88 ± 0.53^c^
300	64.21 ± 0.26^b^	651.01 ± 0.38^d^
400	72.44 ± 0.46^a^	177.08 ± 0.47^e^

*Note:* Means with different letters are significantly different within each column (*p* < 0.05).

Abbreviations: IC_50_, inhibitory concentration; RSA, free radical scavenging activity.

**FIGURE 2:**
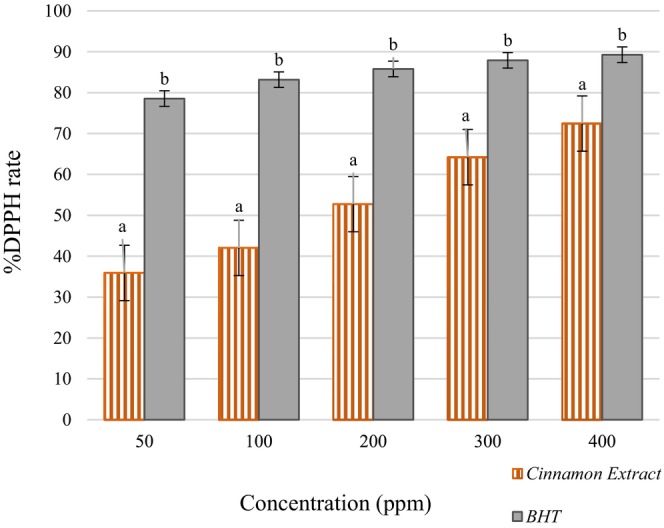
Comparison of the effects of different concentrations of BHT and cinnamon extract on DPPH free radical inhibition. Means with different letters are significantly different (*p* < 0.05). BHT, butylated hydroxytoluene; DPPH, diphenyl picrylhydrazyl.

The comparison of the effects of different concentrations of BHT and cinnamon extract on the inhibition of DPPH free radicals is presented in Figure 2. The results indicate a significant difference between the amounts of BHT and cinnamon extract at the same concentrations (*p* < 0.05). As the concentration of the extract increases, its ability to inhibit free radicals also increases, approaching the inhibitory capacity of BHT. Higher extract concentrations, such as 300 and 400 ppm, showed good free radical inhibition ability (64.21% and 72.44%, respectively) compared to BHT. These levels are comparable to the performance of BHT, indicating that at these concentrations, cinnamon extract can effectively neutralize free radicals similarly to the synthetic antioxidant. This suggests that cinnamon extract, particularly at higher concentrations, could be a natural alternative to BHT for antioxidant purposes, providing similar benefits in terms of free radical inhibition.

### 3.4 FRAP of Cinnamon Extract

In order to enhance the assessment of antioxidant potential, this study additionally utilized the FRAP method to quantify the antioxidant capacity of cinnamon bark ( *Cinnamomum zeylanicum* ). Table 3 illustrates a significant difference in all concentrations compared to the 500 ppm concentration (*p* < 0.05). As the concentration of extract increased, the FRAP percentage rose from 0.49 to 1.80, except at the 2000 ppm concentration, where there was a decrease in FRAP percentage. This unexpected decrease may suggest a saturation point or interference at that concentration.

**TABLE 3:** %FRAP values at different concentrations of cinnamon extract.

Concentration (ppm)	%FRAP
500	0.49 ± 0.011^d^
1000	1.72 ± 0.030^b^
2000	1.48 ± 0.043^c^
3000	1.76 ± 0.025^ab^
4000	1.80 ± 0.026^a^
TBHQ (100)	1.77 ± 0.025^a^

*Note:* Means with different letters are significantly different (*p* < 0.05).

Abbreviations: FRAP, ferric reducing antioxidant power assay; TBHQ, tertiary butylhydroquinone.

Furthermore, TBHQ, when utilized as a comparative synthetic antioxidant, demonstrated a lower antioxidant capacity at the 4000 ppm concentration compared to other concentrations of the extract. This suggests that cinnamon extract may have higher antioxidant efficacy at higher concentrations, but may require significant amounts to match its effects at lower levels. In a related study, the FRAP values for ethanol and dichloromethane extracts of cinnamon bark were reported as 73.02 and 44.63 mg/g, respectively (Abeysekera, Premakumara, and Ratnasooriya 2013). This difference can be attributed to the solvent's ability to extract different bioactive compounds. Ethanol, a polar solvent, is more effective in extracting a greater amount of phenolic compounds, which are recognized for their potent antioxidant properties. In contrast, dichloromethane, a less polar solvent, results in the extraction of a smaller quantity of antioxidant compounds.

### 3.5 TPC and TTC of Oil Cake

Table 4 shows the levels of TPC and TTC in cake samples with varying concentrations of cinnamon bark extract. The findings indicated a statistically significant variation (*p* < 0.05) in TPC and TTC among the six samples tested with different extract concentrations. It was observed that an increase in cinnamon extract in the cakes led to a direct increase in TPC and TTC levels in all samples, except for the 0.15% TTC concentration, where a reduction in tocopherol compounds was noted compared to the 0.1% concentration. Consequently, the samples with a 0.25% concentration exhibited the highest TPC and TTC levels (19.47 and 15.01 mg/mL, respectively) compared to the control sample (3.35 and 1.35 mg/mL, respectively).

**TABLE 4:** Amounts of TPC and TTC in oil cake samples.

Cinnamon extract	TPC (mg/mL)	TTC (mg/mL)
Control sample	3.35 ± 0.06^f^	1.35 ± 0.03^f^
0.05%	10.25 ± 0.03^e^	7.39 ± 0.02^e^
0.10%	12.84 ± 0.04^d^	11.73 ± 0.03^c^
0.15%	17.07 ± 0.02^c^	10.41 ± 0.04^d^
0.20%	18.91 ± 0.08^b^	13.58 ± 0.05^b^
0.25%	19.47 ± 0.04^a^	15.01 ± 0.04^a^

*Note:* Means with different letters are significantly different within each column (*p* < 0.05).

Abbreviations: TPC, total phenolic compounds; TTC, total tocopherol compounds.

In another study, the TPC values for the hydroalcoholic extract of evening primrose oil cakes were 659.51 mg GAE/g, and for the aqueous extract, 229.63 mg GAE/g. These high TPC values can be attributed to the richness of phenolic compounds in evening primrose oil cake, known for its antioxidant properties. Hydroalcoholic extraction is particularly effective in extracting a wide range of bioactive compounds, resulting in higher TPC values than aqueous extraction (Peschel et al. 2007). For sesame cake extracts (concentration 1000 μg/mL), the measured TPC was reported as 27.98 mg/g in the ethanol/water extract and 22.03 mg/g in the aqueous extract, respectively (Esmaeilzadeh Kenari, Mohsenzadeh, and Amiri 2014). The higher TPC for the ethanol extract is consistent with the idea that polar solvents like ethanol can extract more phenolic compounds compared to water (Gülçın et al. 2003). In this study, the TPC values in cakes enriched with cinnamon bark extract reached a maximum of 19.47 mg/mL at a concentration of 0.25%. This amount is lower than the TPC values observed in the studies by Peschel et al. (2007) and Esmaeilzadeh Kenari, Mohsenzadeh, and Amiri (2014). The differences can be attributed to several factors. One likely reason is the reduction in the bioavailability of phenolic compounds due to thermal degradation during baking or interactions with other cake ingredients. In other studies, concentrated phenolic content was achieved through the utilization of direct plant extracts. The inclusion of fats, sugars, and proteins in the cake may disrupt the release or stability of phenolic compounds during the analytical process. Another reason could be the relatively low concentration of cinnamon extract (0.1%–0.25%) compared to the higher concentrations of extract used in other studies.

Regarding TTC, while there are no direct comparisons with tocopherol levels in studies as mentioned above, our findings indicate a strong tocopherol content at higher cinnamon extract concentrations (with a maximum of 15.01 mg/mL at 0.25%). This suggests that cinnamon bark extract may also be a valuable source of tocopherols, contributing to its overall antioxidant activity.

### 3.6 Free Radical Scavenging Activity of DPPH of Oil Cake

Table 5 presents the DPPH free radical inhibition activity in cake samples containing various concentrations of cinnamon bark extract. According to the data, significant differences were observed in the DPPH free radical inhibition activity across different percentages and concentrations of extracts (*p* < 0.05). As the concentration of the extract increased, so did the %RSA, indicating a positive correlation between cinnamon extract concentration and antioxidant activity. In this study, the 400 ppm concentration exhibited the highest level of free radical inhibition. Specifically, the highest %RSA was 37.58%, associated with a 400 ppm concentration of 0.25% cinnamon extract. This finding indicates that, at this concentration, the extract demonstrated the highest efficacy in neutralizing free radicals. The results indicated that lower concentrations are inadequate for maximizing the antioxidant effects of cinnamon extract.

**TABLE 5:** DPPH free radical scavenging activity in oil cake samples.

Cinnamon extract	RSA%				
	50 ppm	100 ppm	200 ppm	300 ppm	400 ppm
Control sample	0.25 ± 0.05^Ef^	0.37 ± 0.02^Df^	0.87 ± 0.04^Cf^	1.12 ± 0.03^Bf^	1.49 ± 0.04^Af^
0.05%	2.42 ± 0.05^Ee^	3.36 ± 0.04^De^	3.49 ± 0.04^Ce^	4.01 ± 0.03^Be^	7.22 ± 0.05^Ae^
0.10%	6.58 ± 0.04^Ed^	9.72 ± 0.07^Dd^	9.93 ± 0.05^Cd^	10.71 ± 0.08^Bd^	12.86 ± 0.07^Ad^
0.15%	10.32 ± 0.03^Dc^	10.08 ± 0.05^Ec^	11.45 ± 0.05^Cc^	13.67 ± 0.04^Bc^	18.91 ± 0.06^Ac^
0.20%	18.27 ± 0.04^Db^	19.09 ± 0.04^Cb^	18.31 ± 0.06^Db^	19.42 ± 0.02^Bb^	22.32 ± 0.03^Ab^
0.25%	22.91 ± 0.07^Ea^	29.98 ± 0.06^Da^	30.64 ± 0.05^Ca^	35.95 ± 0.04^Ba^	37.58 ± 0.03^Aa^

*Note:* Means with different lowercase letters (a, b, c, d, and e) are significantly different within each column (*p* < 0.05), and means with different uppercase letters (A, B, C, D, E, and F) are significantly different within each row (*p* < 0.05).

Abbreviations: DPPH, diphenyl picrylhydrazyl; RSA, free radical scavenging activity.

In a related study, the DPPH free radical scavenging activity (EC_50_ value) of green tea sponge cakes at concentrations of 0%, 10%, 20%, and 30% were 31.81, 12.86, 0.11, and less than 0.01 mg/mL, respectively (Lu et al. 2010). This indicates that green tea sponge cakes exhibit a potent scavenging activity as concentrations increase, aligning with the observed trend in cinnamon extract. Additionally, the free radical inhibition percentage of sesame cake was measured for ethanol/water extract (18.905%–76.525%) and aqueous extract (8.06%–30.03%) (Esmaeilzadeh Kenari, Mohsenzadeh, and Amiri 2014). Another study reported DPPH values for hydroalcoholic and water extracts of evening primrose cake as 31.91% and 61.58%, respectively (Peschel et al. 2007). This comparison emphasizes that different extraction methods and plant materials significantly influence the antioxidant potential, reinforcing the effectiveness of specific extraction techniques. Dhillon and Amarjeet (2013), evaluated the DPPH free radical inhibition percentage in bread samples with varying percentages of cinnamon powder, concluding that higher levels of cinnamon powder resulted in increased DPPH free radical inhibition. This value increased from 4.27% in the control sample to 27.67% in the sample containing 4% (Dhillon and Amarjeet 2013). This supports the findings of the current study, indicating that higher concentrations of cinnamon contribute to increased free radical inhibition. Variability in results across different studies can be attributed to factors such as the type of extract, extraction method, and the food matrix, which affects the availability and stability of antioxidant compounds.

### 3.7 Amount of FRAP of Oil Cake

The antioxidant power of FRAP iron reduction in cake samples with different concentrations of cinnamon bark extract is presented in Table 6. According to the results, there is no significant difference between the control sample at the concentration of 500 ppm and the sample at 1000 ppm. However, there is a significant difference with other concentrations in this group. The FRAP percentage increases with increasing extract concentration. The highest FRAP value among the samples was observed at a concentration of 4000 ppm in the 0.25% sample (1.765%), while the lowest was in the control group at 500 ppm (0.007%). The overall trend indicates that increasing the concentration of cinnamon bark extract enhances its ability to reduce iron ions, thus indicating stronger antioxidant properties. In a study by Esmaeilzadeh Kenari, Mohsenzadeh, and Amiri (2014), the FRAP values of sesame cake for ethanol/water extract, aqueous extract, and methanol/water extract were measured to be 0.16–132, 0.0955–0.460, and 0.185–0.931 mg/mL, respectively (Esmaeilzadeh Kenari, Mohsenzadeh, and Amiri 2014). These values indicate varying antioxidant capabilities depending on the type of extract and solvent used, similar to the results observed in cinnamon extract.

**TABLE 6:** %FRAP values in oil cake samples.

Cinnamon extract	%FRAP				
	500 ppm	1000 ppm	2000 ppm	3000 ppm	4000 ppm
Control sample	0.007 ± 0.05^Ed^	0.011 ± 0.03^Fdc^	0.014 ± 0.01^Fc^	0.028 ± 0.03^Eb^	0.043 ± 0.01^Fa^
0.05%	0.083 ± 0.06^De^	0.094 ± 0.04^Ed^	0.169 ± 0.04^Ec^	0.266 ± 0.07^Db^	0.438 ± 0.13^Ea^
0.10%	0.219 ± 0.02^Ce^	1.366 ± 0.03^Dd^	1.439 ± 0.15^Bc^	1.658 ± 0.03^Cb^	1.673 ± 0.04^Da^
0.15%	0.418 ± 0.08^Bd^	1.648 ± 0.07^Cc^	1.735 ± 0.04^Aa^	1.730 ± 0.06^Aab^	1.724 ± 0.02^Cb^
0.20%	0.421 ± 0.05^Be^	1.667 ± 0.02^Bc^	1.391 ± 0.08^Dd^	1.739 ± 0.03^Ab^	1.752 ± 0.06^Ea^
0.25%	0.435 ± 0.01^Ae^	1.701 ± 0.11^Ab^	1.426 ± 0.04^Cd^	1.697 ± 0.12^Bcb^	1.765 ± 0.09^Aa^

*Note:* Means with different lowercase letters (a, b, c, d, and e) are significantly different within each row (*p* < 0.05), and means with different uppercase letters (A, B, C, D, E, and F) are significantly different within each column (*p* < 0.05).

Abbreviation: FRAP, ferric reducing antioxidant power assay.

### 3.8 Physicochemical Properties of Oil Cake

Table 7 presents the physicochemical analysis of oil cake samples that incorporate different concentrations of cinnamon bark extract ( *Cinnamomum zeylanicum* ). The pH values across the samples remained consistent, ranging between 7.41 and 7.49, with no significant differences observed. However, the moisture content in the samples containing cinnamon extract was notably higher than in the control, showing a significant increase in the samples with 0.15% and 0.25% cinnamon extract (*p* < 0.05). Cinnamon extract may have hygroscopic properties, allowing it to attract and retain moisture, thus resulting in increased moisture content in the final product.

**TABLE 7:** Physicochemical properties of oil cake samples with different concentrations of cinnamon extract.

Parameters	Samples					
	Control sample	0.05%	0.10%	0.15%	0.20%	0.25%
pH	7.45 ± 0.93^a^	7.43 ± 0.81^a^	7.42 ± 0.31^a^	7.41 ± 0.13^a^	7.46 ± 0.66^a^	7.49 ± 0.23^a^
Moisture (%)	23.32 ± 1.53^cb^	26.06 ± 0.90^ab^	24.32 ± 1.84^ab^	27.46 ± 2.53^a^	26.66 ± 2.83^ab^	23.39 ± 1.48^b^
Water Activity	0.70 ± 0.02^a^	0.64 ± 0.01^b^	0.69 ± 0.03^a^	0.69 ± 0.01^a^	0.68 ± 0.03^ab^	0.67 ± 0.01^ab^
Protein (%)	10.05 ± 0.35^d^	10.09 ± 0.16^cd^	10.13 ± 0.21^bc^	10.16 ± 0.20^ab^	10.16 ± 0.40^ab^	10.19 ± 0.12^a^
Fat (%)	13.46 ± 0.29^a^	13.50 ± 0.26^a^	13.17 ± 0.06^ab^	12.87 ± 0.22^b^	13.05 ± 0.09^b^	13.03 ± 0.11^b^
Carbohydrate (%)	42.65 ± 1.03^a^	42.74 ± 0.70^a^	40.99 ± 1.24^bc^	40.04 ± 0.82^c^	41.88 ± 0.48^ab^	39.82 ± 0.49^c^
Total Ash (%)	2.40 ± 0.17^a^	2.44 ± 0.16^a^	2.49 ± 0.43^a^	2.27 ± 0.13^a^	2.30 ± 0.19^a^	2.29 ± 0.27^a^
Acid Insoluble Ash (%)	0.16 ± 0.03^c^	0.36 ± 0.02^bc^	0.68 ± 0.05^b^	0.63 ± 0.09^b^	0.66 ± 0.51^b^	1.18 ± 0.19^a^
Length (mm)	22.49 ± 3.52^a^	23.64 ± 1.71^a^	23.70 ± 6.51^a^	23.49 ± 3.17^a^	22.80 ± 5.94^a^	23.15 ± 1.99^a^
Width (mm)	20.83 ± 5.21^bc^	17.17 ± 1.33^b^	21.10 ± 3.49^bc^	24.08 ± 2.48^ac^	25.28 ± 0.25^ac^	27.44 ± 0.78^a^
Depth (mm)	18.45 ± 4.84^a^	17.25 ± 1.65^a^	21.90 ± 3.03^a^	22.70 ± 3.88^a^	21.69 ± 5.99^a^	23.04 ± 2.84^a^

*Note:* Means with different letters are significantly different within each row (*p* < 0.05).

In the study by Amiri and Eshaghi (2024), which focused on the production of cinnamon breakfast cakes with varying percentages of cinnamon essence, results similar to our study were obtained. They reported that the pH of the prepared cakes did not show a significant difference compared to the control sample (*p* > 0.05), with the pH of the breakfast cakes measured between 7.39 and 7.49. Additionally, the moisture content of the cakes that included cinnamon essence showed a significant effect (*p* < 0.05), with the highest moisture content reported in the treatments containing 0.1% and 0.2% cinnamon essence (Amiri and Eshaghi 2024). The water activity values of the oil cake samples showed that as the concentration of cinnamon extract increased, the water activity decreased in comparison to the control sample. A notable distinction was identified between the control sample and those containing 0.05%, 0.10%, and 0.15% cinnamon extract. The constituents found in cinnamon extract have the potential to interact with water molecules, thereby diminishing the quantity of unbound water present in the cake. This interaction results in a lowered water activity level. In a study on cinnamon cake, the pH value was found to be 7.03. The moisture content measured in the study ranged between 22.86% and 32.75%, while the water activity values varied from 0.84 to 0.89 (Mona, Thanaa, and Meranda 2016). The protein content in the cake samples with varying concentrations of cinnamon extract ranged from 10.05% in the control sample to 10.19% in the sample with 0.25% extract. Moreover, the sample containing 0.25% extract showed a significant difference when compared to the control, as well as the 0.05% and 0.10% samples (*p* < 0.05). In the study by Dhillon and Amarjeet (2013), it was concluded that with an increase in cinnamon powder, the protein percentage of the bread samples increased from 8.06% for the control sample to 8.37% for the 4% sample (Dhillon and Amarjeet 2013). Additionally, in the study by Mona, Thanaa, and Meranda (2016), the protein content of cake samples containing 3% cinnamon was measured at 7.88% (Mona, Thanaa, and Meranda 2016). The incorporation of cinnamon could potentially improve the solubility of proteins within the cake batter, resulting in an increased detectable protein concentration. Cinnamon can interact with other ingredients, promoting protein binding and increasing the overall protein content in the final product.

The measured fat percentage for all samples, except the 0.05% sample, decreased compared to the control sample. The fat content was 13.46% for the control sample and 13.03% for the 0.25% sample. Cinnamon may have emulsifying properties that affect fat dispersion in the cake, potentially resulting in reduced fat retention. In the study by Lu et al. (2010) on sponge cake made with green tea powder replacing cake flour, the measured fat percentage was 10.21% for the control sample and 10.19% for the 30% sample (Lu et al. 2010). As the percentage of extract increased, the carbohydrate content decreased from 42.65% in the control sample to 39.82% in the sample containing 0.25% extract. The addition of cinnamon extract may dilute the carbohydrate content, resulting in a decrease in the overall percentage of carbohydrates in the cake. In the study by Hafez (2012), the carbohydrate content of cake samples enriched with marjoram extract showed that with an increase in extract level, the carbohydrate content increased, from 50.4% in the control sample to 66.61% in the 3% sample containing marjoram extract (Hafez 2012). No significant differences were found in the total ash content across the various samples in this study (*p* < 0.05). Conversely, there was a direct correlation between the percentage of extract and the increase in acid‐insoluble ash content in the cakes, rising from 0.16% in the control sample to 1.18% in the sample with 0.25% extract. The presence of minerals in the cinnamon extract can contribute to an increase in the acid‐insoluble ash content, as these minerals are not soluble in acid and contribute to the overall ash content in the cakes. In Hafez's (2012) research, the analysis of total ash content in cake samples supplemented with marjoram extract indicated that higher levels of the extract corresponded to an increase in total ash content. The variations in physicochemical properties upon adding cinnamon extract can be attributed to the unique chemical composition and interactions of cinnamon with other ingredients. Understanding these effects can help optimize formulations for desired texture and quality in food products.

In addition, the dimensions of oil cakes with varying concentrations of cinnamon extract, including length, width, and depth, were analyzed using a Texture Analyzer Meter. In most cases, no notable differences were detected. Therefore, enriching oil cakes with aqueous cinnamon extract generally does not affect the length, width, or depth. Altering the formulation of cake samples can significantly influence the texture characteristics associated with physicochemical or structural phenomena (Soukoulis, Gaiani, and Hoffmann 2018). The study provides insightful data on the effects of cinnamon extract on the physicochemical properties of oil cake samples.

### 3.9 TPA of Oil Cake

The changes in textural characteristics of oil cake samples by adding cinnamon bark aqueous extract ( *Cinnamomum zeylanicum* ) in different concentrations are shown in Table 8. The TPA of the measured cake indicated that increasing the level of cinnamon extract in the oil cake decreased its hardness, making the cake softer. Specifically, the hardness of the control sample decreased from 3816.33 to 2853.00 g in the sample with 0.25% extract, demonstrating an improvement in texture and hardness with adding cinnamon extract. The reduction in hardness is likely due to the added moisture and water‐binding properties of the cinnamon extract. Our results are consistent with the findings of Majzoobi et al. (2014), who observed that the hardness of cakes decreased with an increasing percentage of resistant corn starch (Majzoobi et al. 2014). Similarly, Salehi et al. (2016) showed that the hardness of cake samples became softer with higher levels of button mushroom powder (Salehi et al. 2016). Different studies have reported varying results. The incorporation of varying quantities of guar gum into carrot sponge cake was observed to enhance the firmness of the cake's texture (Salehi and Kashaninejad 2021). In the research conducted by Nakov et al. (2020), it was observed that an increase in the quantity of grape pomace powder resulted in a greater hardness of the cake (Nakov et al. 2020). In another study, Lu et al. (2010) found that the texture of sponge cake becomes harder with higher levels of green tea powder. These differences in results can be attributed to the type of material added (Lu et al. 2010).

**TABLE 8:** Textural properties of oil cakes with different concentrations of cinnamon extract.

Parameters	Cinnamon extract					
	Control sample	0.05%	0.10%	0.15%	0.20%	0.25%
Hardness (g)	3816.33 ± 8.89^a^	3133.67 ± 8.14^ac^	3042.00 ± 0.95^ac^	2986.33 ± 7.02^ac^	2964.00 ± 3.12^ac^	2853.00 ± 2.98^bc^
Adhesiveness (mj)	0.20 ± 0.26^bc^	0.40 ± 0.17^ac^	0.86 ± 0.49^a^	0.34 ± 0.17^bc^	0.47 ± 0.31^ac^	0.23 ± 0.15^bc^
Cohesiveness	0.73 ± 0.13^a^	0.78 ± 0.02^a^	0.62 ± 0.06^a^	0.78 ± 0.36^a^	0.58 ± 0.02^a^	0.56 ± 0.02^a^
Resilience	0.24 ± 0.02^a^	0.25 ± 0.02^ad^	0.22 ± 0.15^ba^	0.18 ± 0.01^cb^	0.19 ± 0.02^db^	0.20 ± 0.03^cd^
Fracturability (g)	3816.33 ± 0.88^a^	3133.67 ± 0.81^a^	3129.00 ± 0.65^a^	3120.00 ± 0.66^a^	2964.00 ± 3.12^a^	2889.33 ± 1.65^a^
Springiness (mm)	9.73 ± 0.61^a^	10.34 ± 0.06^a^	10.91 ± 3.32^a^	11.39 ± 0.44^a^	11.81 ± 0.30^a^	12.11 ± 0.14^a^
Gumminess (g)	276.46 ± 0.45^a^	246.26 ± 0.67^ab^	199.36 ± 2.93^bc^	176.80 ± 0.44^c^	172.16 ± 1.17^c^	186.56 ± 5.25^bc^
Chewiness (mj)	284.97 ± 0.38^a^	267.63 ± 0.54^ab^	115.57 ± 0.69^c^	155.73 ± 2.85^c^	177.37 ± 0.56^bc^	256.87 ± 0.45^ab^

*Note:* Means with different letters are significantly different within each row (*p* < 0.05).

Adhesiveness is a surface property that depends on the combined effect of adhesion forces. It is the amount of work required to overcome the forces of attraction between the surface of the food and the surfaces in contact with the food in the mouth (e.g., tongue, palate, teeth) (Huang et al. 2007). The adhesiveness values of the oil cake texture remained consistent and showed no significant differences with the increasing percentages of cinnamon extract. Only the sample with 0.10% extract exhibited a significant difference when compared to the control samples and those with 0.15% and 0.25% extracts. However, all measured values for this parameter were higher than that of the control sample. This might be due to the minimal effect of cinnamon on surface interactions. Slima et al. (2021), found that using the new polysaccharide *Lepidium sativum* in cake formulation increased cake adhesion (Slima et al. 2021). Cohesiveness indicates the internal strength of the food structure, reflecting the ability of a substance to stick to its particles (Salehi and Kashaninejad 2021). It refers to the intra‐tissue connections that make up the food item's structure, with values closer to one indicating greater internal resistance to applied forces and deformation (Ben Slima et al. 2022). One reason for the consistency of the cake may be due to moisture or its circular cells (Slima et al. 2021).

The results of TPA in our study showed a slight decrease in the consistency of the cake with an increase in the level of cinnamon extract in most of the samples. None of the samples were significantly different from each other. Additionally, the resilience of the cakes decreased with the increase in extract percentage. The slight decrease in cohesiveness and resilience with increasing cinnamon extract could be due to changes in the cake's internal structure, specifically the moisture distribution and cell wall breakdown. These effects reduce the cake's ability to resist deformation, resulting in a more fragile texture. Our results were consistent with the study by Lu et al. (2010), which reported a reduction in cohesiveness and resilience values in sponge cake samples with an increase in green tea powder levels (Lu et al. 2010). Another study measuring the TPA of cake enriched with different percentages of quince fruit powder showed that with an increase in powder level, the cohesiveness and resilience values of the cake samples decreased (Salehi and Kashaninejad 2017). Despite the differences in the enrichment material and the type of cake in the compared studies, their results were consistent with those obtained in our study.

The cakes exhibited a reduction in resilience as the percentage of extract increased. Fracturability decreased with a higher percentage of extract in the cakes, but no significant difference was observed between the samples (*p* < 0.05). The decrease in fracturability suggests that the cake becomes less brittle with more cinnamon extract, possibly due to improved hydration and a softer structure. However, the difference was not statistically significant. In a study by Lee, Sim, and Chun (2009) on chiffon cake made with mulberry powder, no difference in fracturability was found between samples with different percentages of berry powder (Lee, Sim, and Chun 2009). Additionally, a study conducted by Jo et al. (2010) on sponge cakes made with Lentinus edodes powder found that the fracturability of the sponge cake samples was lower than that of the control cakes (Jo et al. 2010), which was consistent with our study. The springiness property measures the recovery between the first and second pressure, indicating the elasticity of the food sample. From the sensory perspective, springiness or elasticity refers to the return of the deformed material to its original condition (without deformation) after the removal of the chewing force (Salehi et al. 2016). However, TPA results showed that increasing the level of cinnamon extract did not significantly affect the springiness and elasticity of the cake. Nevertheless, the increase in the percentage of the extract was directly related to the improvement in the cake's properties, indicating the potential for adding cinnamon extract to the oil cake. Similar results were reported by Ben Slima et al. (2022) when applying the new polysaccharide *Lepidium sativum* in cake formulations (Ben Slima et al. 2022). Additionally, another study conducted by Salehi and Kashaninejad (2021) indicated that the springiness of the cake enhanced as the concentration of guar gum was elevated. However, Lu et al. (2010) reported that, increasing the level of green tea powder in the cake decreased its springiness properties. Therefore, it seems that the difference in the results was related to the type of added substance.

Gumminess and chewiness indicate the energy required to break down food during swallowing. From a sensory perspective, gumminess and chewiness refer to the energy needed to break down semi‐solid food to obtain a ready‐to‐swallow product (Salehi and Kashaninejad 2021). The TPA results in this study showed a decrease in the gumminess and chewiness properties of the oil cake samples compared to the control sample, with an increase in the level of cinnamon extract. These results were consistent with those of Salehi and Kashaninejad (2021) study regarding adding guar gum to cake. Additionally, Majzoobi et al. (2014) reported that increasing the percentage of resistant starch in cake decreased its chewiness (Majzoobi et al. 2014). However, Lu et al. (2010) found that the gumminess and chewiness of the cake increased with the level of green tea powder (Lu et al. 2010). Another study showed that cake enriched with different percentages of quince fruit powder had increased gumminess and chewiness properties compared to the control sample (Salehi and Kashaninejad 2017). These results highlight that the impact of cinnamon extract on cake texture primarily results from its ability to retain moisture and weaken the structural matrix, leading to a softer, less dense texture. Differences in texture across various studies can often be attributed to the specific properties of the added materials and their interactions with the cake's ingredients. Due to the difference in the type of enrichment material and the type of cake, their results were inconsistent with the results obtained in this study.

### 3.10 Sensory Evaluation of Oil Cake

Sensory evaluation of food products is usually based on consumers' visual and sensory perceptions. Thus, sensory analysis is essential for assessing the acceptability of foods during the development of new products (Nakov et al. 2020). Sensory evaluation of cake quality is mainly based on personal judgment and subjective qualitative assessment, which can reflect consumer preferences (Jeddou et al. 2017). Table 9 evaluates the sensory characteristics of oil cakes enhanced with varying concentrations of cinnamon extract.

**TABLE 9:** Sensory evaluation of oil cakes containing different concentrations of cinnamon extract.

Parameters	Cinnamon extract					
	Control sample	0.05%	0.10%	0.15%	0.20%	0.25%
Color	7.20 ± 1.47^a^	7.21 ± 1.28^a^	7.25 ± 1.12^a^	7.45 ± 1.54^a^	7.75 ± 1.07^a^	7.85 ± 1.27^a^
Odor	5.80 ± 1.85^c^	7.15 ± 1.46^b^	7.45 ± 1.19^b^	7.60 ± 1.23^ab^	7.50 ± 1.10^ab^	8.30 ± 0.80^a^
Taste	7.00 ± 1.95^ab^	6.80 ± 1.54^b^	7.35 ± 1.27^ab^	7.50 ± 1.32^ab^	7.75 ± 1.33a	7.65 ± 1.14^ab^
Texture	7.00 ± 2.25^b^	7.25 ± 1.44^ab^	7.05 ± 1.47^b^	7.80 ± 0.95^ab^	8.00 ± 1.17^a^	7.70 ± 1.30^ab^
Overall acceptability	6.95 ± 1.64^b^	7.25 ± 1.21^ab^	7.40 ± 1.14^ab^	7.70 ± 0.98^a^	7.95 ± 0.95^a^	7.95 ± 0.69^a^

*Note:* Means with different letters are significantly different within each row (*p* < 0.05).

One of the most essential features of food products is the color factor (Martinez‐Giron, Figueroa‐Molano, and Ordóñez‐Santos 2017). In this study, the color of the samples with different percentages of extract did not show a significant difference compared to each other (*p* < 0.05). However, the results indicated a direct relationship between the increase in extract percentage and the color of the oil cakes. With the increase in extract percentage, the color score of the oil cakes improved. The average color factor score for the control sample was 7.20, and for the 0.25% sample, it reached 7.85. The analysis of the odor factor for different samples showed a significant difference between the control sample and other samples (*p* < 0.05). Among the different samples, the highest cinnamon extract odor score belonged to the 0.25% sample (8.30). The taste of oil cake samples containing different concentrations of cinnamon extract, except for the 0.05% and 0.20% samples, did not show a significant difference. The highest taste score belonged to the 0.20% sample (7.75). All samples had higher texture scores compared to the control sample, and the only significant difference was related to the 0.20% sample (*p* < 0.05). Regarding the overall acceptability, the control sample displayed a significant difference compared to the oil cake samples containing 0.15%, 0.20%, and 0.25% extract (*p* < 0.05). There was a direct relationship between the increase in extract and the overall acceptability of the cake samples. The highest overall acceptability scores belonged to the 0.20% and 0.25% extract samples (7.95). Cinnamon essential oil provides a unique flavor characteristic of the cinnamon spice. When incorporated into cake batter, it enhances the overall flavor profile of the cake, imparting a warm and aromatic quality (Hiregoudar, Revanna, and Mamatha 2023). Consequently, essential oils often influence consumer preferences and are widely used in significant amounts for flavoring, thanks to their pleasant aroma and distinct taste.

A study on cakes supplemented with varying levels of marjoram found no notable differences in color, texture, or overall acceptability ratings between samples with and without marjoram extract. However, there was a significant difference in taste and odor between the control and other concentrations (Hafez 2012). Saber's (2019) study on cakes and bread enriched with different percentages of cinnamon also showed that adding cinnamon affected all sensory characteristics compared to control samples. Increasing the cinnamon percentage resulted in higher organoleptic scores for both cakes and bread. These findings were consistent with the sensory evaluation results from our study (Saber 2019).

## 4 Conclusion

Cinnamon extract ( *Cinnamomum zeylanicum* ), due to its phenolic compounds, possesses good antioxidant properties. As the concentration of the extract increased, the antioxidant activity of the oil cake also improved. It seems that extracted cinnamon can be used as a natural antioxidant in the food industry. HPLC analysis of the aqueous cinnamon extract identified cinnamaldehyde as the primary compound, making up 30.5% of the total peak area. Significant amounts of eugenol, cinnamic acid, and coumarin were also detected, along with minor components. The chromatogram clearly outlined the retention times and peak intensities of these bioactive compounds, providing a detailed chemical profile of the extract. The oil cake enriched with cinnamon extract exhibited various physicochemical properties with positive outcomes. The pH of the different samples did not show significant differences. The increase in extract concentration resulted in a reduction of water activity in the samples compared to the control sample. Increasing the percentage of cinnamon extract led to an increase in the protein content of the cake, a decrease in fat, and a reduction in carbohydrates in most samples compared to the control sample. Among the TPA parameters, a decrease in hardness and an increase in springiness were considered two important texture parameters with favorable results for enriching the oil cake with cinnamon extract. The highest score for overall acceptability belongs to the 0.20% and 0.25% extract samples. Therefore, this situation can provide a suitable opportunity to ensure consumer health.

## Author Contributions

**Banafshe Bordbar Lomer:** investigation (equal), methodology (equal), project administration (equal), writing – review and editing (equal). **Fatemeh Ghannadiasl:** methodology (equal), supervision (equal), writing – original draft (equal), writing – review and editing (equal).

## Ethics Statement

This study was approved by the Institutional Review Board of the University of Mohaghegh Ardabili.

## Conflicts of Interest

The authors declare no conflicts of interest.

## Data Availability

The datasets generated for this study are available upon reasonable request to the corresponding author.
